# Iodine Intake through Processed Food: Case Studies from Egypt, Indonesia, the Philippines, the Russian Federation and Ukraine, 2010–2015

**DOI:** 10.3390/nu9080797

**Published:** 2017-07-26

**Authors:** Jacky Knowles, Frits van der Haar, Magdy Shehata, Gregory Gerasimov, Bimo Bimo, Bettina Cavenagh, Cherry C. Maramag, Edward Otico, Doddy Izwardy, Rebecca Spohrer, Greg S. Garrett

**Affiliations:** 1 Large Scale Food Fortification Initiative, Global Alliance for Improved Nutrition, 1202 Geneva, Switzerland; jacky@jackyknowlesconsultancy.com (J.K.); becca@springaccelerator.org (R.S.); 2 Iodine Global Network, Ottawa, ON K1E 3E6, Canada; fvander@emory.edu (F.v.d.H.); greg.gerasimov@gmail.com (G.G.); 3 Nutrition/Food Technology, United Nations, World Food Programme, Cairo, Egypt; magdy.shehata@outlook.com; 4 Large Scale Food Fortification Initiative, Global Alliance for Improved Nutrition, Jakarta 12950, Indonesia; bimo.jkt@gmail.com; 5 PT Clarity Research Indonesia, Jakarta 12920, Indonesia; bettina@clarity.co.id; 6 Research and Program Unit, Nutrition Center of the Philippines, Muntinlupa City 1780, Philippines; cmaramag@ncp.org.ph; 7 Large Scale Food Fortification Initiative, Global Alliance for Improved Nutrition, Manila 1000, Philippines; edwardpotico@yahoo.com; 8 Community Nutrition, Indonesia Ministry of Health, Jakarta 12950, Indonesia; izwardydoddy@gmail.com

**Keywords:** iodised salt, processed foods, iodine deficiency disorders

## Abstract

The current performance indicator for universal salt iodisation (USI) is the percentage of households using adequately iodised salt. However, the proportion of dietary salt from household salt is decreasing with the increase in consumption of processed foods and condiments globally. This paper reports on case studies supported by the Global Alliance for Improved Nutrition (GAIN)-UNICEF USI Partnership Project to investigate processed food industry use of adequately iodised salt in contrasting national contexts. Studies were conducted in Egypt, Indonesia, the Philippines, the Russian Federation, and Ukraine. In all cases, the potential iodine intake from iodised salt in selected food products was modelled according to the formula: quantity of salt per unit of food product × minimum regulated iodine level of salt at production × average daily per capita consumption of the product. The percent of adult recommended nutrient intake for iodine potentially provided by the average daily intake of bread and frequently consumed foods and condiments was from 10% to 80% at the individual product level. The potential contribution to iodine intake from the use of iodised salt in the processed food industry is of growing significance. National USI strategies should encourage co-operative industry engagement and include regulatory monitoring of iodised salt use in the food industry in order to achieve optimal population iodine status.

## 1. Introduction

Iodine deficiency hinders cognitive development and growth, and is the single greatest cause of preventable mental impairment in the world. Universal Salt Iodisation (USI), defined as iodisation of all food-grade salt used in the household, by food processing industries and for animal feed [1], is globally accepted as the most cost-effective public health strategy to prevent and control iodine deficiency disorders (IDD) [2].

From 2008 to 2015, the Global Alliance for Improved Nutrition (GAIN) and UNICEF worked in a partnership with a grant from the Bill & Melinda Gates Foundation to intensify business-oriented efforts towards the global elimination of iodine deficiency through USI (“the Partnership Project”). The goal of the Partnership Project was to sustainably improve population-wide iodine intakes, primarily by increasing the supply of, and access to, adequately iodised food-grade salt in 13 countries with: a very high population numbers unprotected from iodine deficiency through lack of access to adequately iodised (≥15 mg/kg) household salt; the lowest percentage of household iodised salt coverage; and/or the greatest potential for sustainably scaling-up efforts through innovative business approaches.

Early on, the Partnership Project recognised that the established performance indicator for USI, household iodised salt coverage, is not an accurate indicator since it does not account for dietary iodine intake from iodised salt in industrially processed foods [3], or the impact of using iodised salt in animal feed.

Salt from manufactured foods including meat, cheese, and bread already constitutes the major share of the salt intake in industrialised countries [4,5]. In low- and middle-income countries (LMIC), consumption of processed foods and condiments has been rising over the past few decades, influenced by income growth, urbanisation, increasing female workforce participation and changing lifestyle practices and choices [6,7]. For example, household expenditure studies in Bangladesh, Nepal, India, Indonesia, and Vietnam have shown that industrially manufactured foods (high and low value added processed foods combined) represent about three quarters of the share of household food purchases in urban areas and more than half in rural areas [6,8,9]. In East and Southern Africa, one third of the purchased food market was highly processed foods, which are more likely to include salt as an ingredient. The relative proportion of this market share increased with income in both rural and urban areas [7]. The referenced literature provides evidence that, as incomes rise, dietary trends diversify beyond staple grains towards fruits, vegetables, animal proteins, dairy, processed products to cook at home, and ready-prepared foods bought away from home.

**Figure 1:**
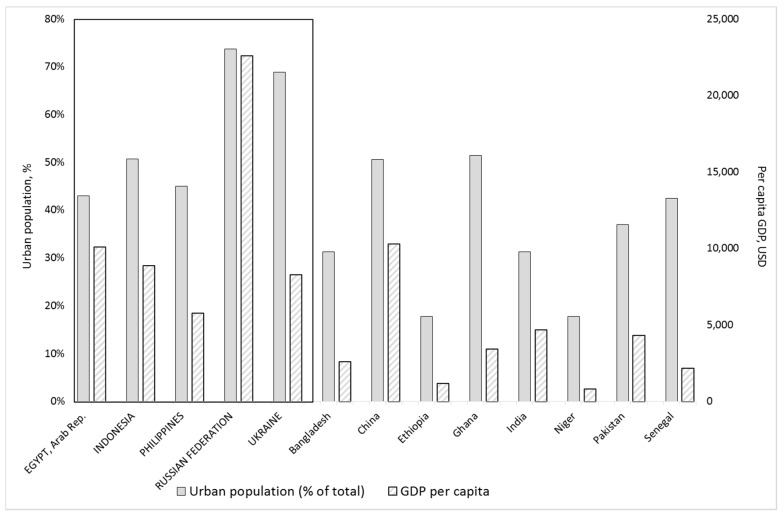
Percentage of population in urban areas (solid fill bars) and per capita gross domestic product (USD) (hashed fill bars) in the Partnership Project countries, 2011. The five countries for which case studies are presented in this paper are listed first and highlighted with a border.

The Partnership Project hypothesised that in some countries, while a significant proportion of population salt intake may be through processed foods and condiments, this may not be iodised due to a lack of: food industry awareness, legislation requiring iodisation of food-industry salt, and/or of clear implementing regulations and regulatory monitoring processes where such legislation exists. Therefore, between 2010 and 2015, GAIN investigated the use of iodised salt in the food industry, including assessment of its current use, identification of obstacles to its use, and modelling the potential impact on dietary iodine intake if it was used. This paper summarises the process and outcome of investigations in four distinct contexts (in relation to country, dietary practices, the legislative situation, the Partnership Project approach, and the food types targeted): Egypt, Indonesia, the Philippines, and the Russian Federation and Ukraine combined. These Partnership Project countries were selected because they had a high proportion of urban populations, combined with a relatively high gross domestic product per capita (Figure 1), factors known to be linked to increased access to high value added processed foods, as described above. Following the situation summaries, we include recommendations on improving policies and practice for iodised salt use and monitoring within the food industry, in order to optimise and sustain population iodine nutrition.

## 2. Materials and Methods

GAIN (along with UNICEF in the Russian Federation and Ukraine) collaborated with national partners and commissioned research in: (i) Egypt, to assess the implementation of existing regulations for the use of iodised salt in the production of Baladi bread at small-scale bakeries throughout the country; (ii) Indonesia and the Philippines, to clarify whether large scale producers of selected processed foods were using iodised salt, how the quality of iodisation was assessed, and industry awareness and understanding of iodised salt regulations; and (iii) the Russian Federation and Ukraine, to review the use of iodised salt in bakery regulations and to support implementation of communication and advocacy efforts to include iodised salt in industrial-scale and artisanal baking of assorted types of bread, with a focus on widely available “social” bread.

The specifics of the research varied by national context and are described further for each case study below. For all country contexts, the potential contribution to population iodine intake was modelled based on a scenario where all salt in the target product was iodised according to the minimum national iodine standard. The following basic formula was used in modelling:

Quantity of salt per unit of food product × minimum regulated iodine level of salt at production × average daily per capita consumption of the product.

## 3. Results

Results are presented for each national case study, providing information on: the context; the investigative process conducted; outcomes of the process, including modelling of iodine intake; and discussion of the implications for national programmes to achieve optimal iodine status.

### 3.1. Egypt-Baladi Bread

Salt iodisation in Egypt started in 1996 with an aim to reduce iodine deficiency among the population. Salt iodisation is now implemented according to mandatory legislation for iodisation of household salt and of salt used in the baking industry (Specification No. 2732-1/2005 issued by the Egyptian organisation of standardisation and quality). Regulations state that salt should be iodised at the level of 30–70 mg/kg potassium iodate (KIO_3_), equivalent to approximately 18–41.5 mg/kg iodine.

Egypt has a national Food Subsidy Program which targets poor and low-income groups. At the time of the survey in 2013, reported below, the subsidy program was made up of two main subsystems: ration cards that offer eligible households specific monthly quotas of subsidised commodities, in particular sugar, edible oil, rice, and tea; and Baladi bread, which was distributed through market outlets on a first-come, first-served basis. Baladi bread was sold at the highly subsidised rate of 5 piastres per loaf, when the real cost of production at that time was approximately 35 piastres according to the average international price of wheat (Personal communication from Dr. Magdy Shehata, World Food Programme (WFP), Cairo). A 2010 WFP survey of consumer behaviour found that respondents from 80% of households in urban areas and 65% of households in rural areas purchased subsidised Baladi bread. Consumption in urban areas was higher because Baladi bread was more easily available and because in some governorates rural households still baked bread at home [10,11]. Subsidised Baladi bread is mandated to be baked using flour fortified with iron and folic acid, and using iodised salt. It is baked in over 24,000 bakeries throughout the country and sold through a network of many thousand outlets (personal communication from Magdy Shehata, WFP Cairo). It was estimated that, on average, Egyptians consumed 380 g flour from bread (equivalent to approximately 475 g bread) per day [10].

Bakeries are monitored by the Ministry of Supply and Internal Trading (MOSIT) and the Ministry of Health and Population (MOHP) to ensure that the bread conforms to quality specification standards. Inspection procedures focus mainly on checking that fortified flour is being used but also include a visual check of available salt packaging to ensure it is labelled as iodised. However, these visual checks may not guarantee that the salt is iodised since packaging and sale of non-iodised salt in bags labelled as iodised has been cited as one of the challenges to achieving USI in Egypt [11]. Where a bakery is found to be using salt in packaging with no indication that it is iodised, a written warning is issued and sometimes the bakery is fined.

GAIN partnered with the WFP to integrate testing of salt iodine into the MOSIT inspections at bakeries using semi-quantitative salt iodine rapid test kits (RTKs), and to record these results in the quality control system database in MOSIT, with the intention of strengthening the government’s inspection capacity and integrating information about bakery use of iodised salt into the Management Information System (MIS) that had been developed to monitor bakery use of fortified flour. As part of the process, MOSIT inspectors were trained in inspecting bakeries for cleanliness, salt storage, the presence of an iodine label or logo on salt packaging, conducting an RTK test, completion of the hard copy inspection form, and data entry into the MIS. A pilot test was conducted, covering 207 randomly-selected bakeries from 25 governorates, to test the planned data collection and reporting system. For the purpose of this first assessment only, MOSIT inspectors included collection of information on the amount of salt used in Baladi bread production and also took bread samples to measure the actual iodine content, with testing conducted at the National Nutrition Institute Laboratory using the Association of Official Analytical Chemists International method 935.14 [12].

The proposed use of results from this inspection process was for the MOHP to follow up on any bread produced using non-iodised salt, investigate whether it was due to non-iodised salt repackaged into bags labelled as iodised, and to develop an appropriate strategic response.

For modelling purposes, the initial salt iodine level was estimated based on a back-calculation from the measured iodine content of the collected bread and bakery-reported salt content of the bread (% salt per unit of flour). This approach estimated the minimum original salt iodine content, since some iodine may have been lost during baking. The number of bakeries selected in each governorate ranged from 2 to 15, with 10 bakeries in the majority of governorates. The number per governorate was selected to provide a similar level of capacity strengthening to each governorate MOSIT team, however it meant that the sample was not proportionally representative of the total number of bakeries by governorate or nationwide.

Seventy six percent of the salt samples (*n* = 158) collected from bakeries in all governorates contained ≥15 mg/kg iodine, as assessed based on the intensity of colour change with the RTK. Using the same method, 23% of salt samples had between 1 and 14.9 mg/kg iodine, and 1% (*n* = 1) was recorded as having no iodine.

The mean iodine content in bread was found to be 0.189 mg/kg, or 18.9 µg iodine per 100 g of bread. See Table 1. The amount of salt used in bread production varied across bakeries from 0.44 g to 0.85 g per 100 g of bread, with an average of 0.67 g/100 g of bread. Using the back-calculation method to estimate iodine in the salt used to produce the bread, a higher number of bread samples (187 or 90% of samples) were found to have been produced using adequately iodised salt (≥15 mg/kg) than was found by RTK; four samples (2%) were found to have been produced with salt with no added iodine (<5 mg/kg), and the remaining 16 bread samples (8%) were found to have been produced with salt containing 5–14.9 mg/kg iodine.

Modelling of expected adult iodine intake from the use of iodised salt in Baladi bread was done based on the measured iodine content of 207 Baladi bread samples, a conservative daily consumption estimate of 400 g bread per adult, and the WHO recommended nutrient intake for iodine (RNI) for non-pregnant, non-lactating adults, of 150 µg [13].

Table 1 indicates that the typical daily consumption of Baladi bread could be contributing an average of 76 µg iodine (50% RNI) to the adult Egyptian diet. The value varied from 36 µg iodine (24% of the adult RNI) in Beni-Suef governorate to 119 µg iodine (80% RNI) in Kalyubia governorate. The derived estimates for the minimum level of iodine in salt used to produce the bread ranged from 16.5 mg/kg to 57.3 mg/kg in these two governorates respectively. The mean (derived) salt iodine estimate was slightly below the national standard of 18 mg/kg iodine (30 mg/kg KIO_3_) in three governorates and above the upper limit of the national standard of 41.5 mg/kg iodine (70 mg/kg KIO_3_) in eight governorates.

The high level of consumption of Baladi bread across different consumer groups in Egypt makes it an optimal food to increase the population’s access to iodised salt, thus increasing dietary iodine intake. The fact that the practice of counterfeit salt labelling in Egypt exists, means regular inspection of the actual iodine content of salt in the bakeries remains a necessary component of monitoring and enforcement to achieve and sustain optimal iodine nutrition. The data reported here indicate that the majority of bakeries are complying with the regulations to use iodised salt, which can be fairly reliably assessed using the RTK.

Where adequately iodised salt is used and bakery-produced Baladi bread is eaten, the modelling suggests it could contribute to approximately half of the recommended daily iodine intake for adults. This is, however, based on some underlying assumptions about the average amount of Baladi bread consumed (which may not be equal in all governorates and/or population groups) and that all bread consumed is sourced from bakeries. In reality, there is evidence that in rural areas of Lower and Upper Egypt and in Frontier governorates, 30%, 40% and 54% of households, respectively, may make their own bread [14]. While many of these households may also purchase some Baladi bread, it cannot be assumed that household members will be consuming the same levels of iodine from bread as found in this modelling exercise. This would depend also on the quality of iodisation of household salt used in home baking, which tends to be poorer in rural areas of these regions [15,16]. The 2014–2015 national iodine survey found that the population of these areas above tends towards poorer, although adequate, iodine status (data from primary school age children in rural Lower and Upper Egypt, no data for Frontier governorates) [16]. The national iodine survey also reported an association between household salt iodine content and iodine status, which could include an expected effect from the use of iodised salt in home-produced Baladi bread.

Results in Table 1, which are only available by governorate (not by urban/rural residence), indicate that, surprisingly, Baladi bread in Upper and Lower Egypt and in the Frontier governorates, contained higher average levels of iodine than in Metropolitan governorates, where access to adequately iodised household salt was highest in the 2014–2015 survey. This finding supports the hypothesis that iodised salt in food products can help reduce inequities in access to dietary salt iodine that might otherwise occur if the only source was adequately iodised household salt.

The recent introduction of a smart card system for subsidised Baladi bread [17,18] permitting access to different quantities of subsidised bread by some measure of socio-economic status, may help target access to the product towards generally poorer, rural, households where it may help counteract the fact that household salt was of poorer iodisation quality. Whether this targeted access to subsidised Baladi bread will be associated with an increase in the proportion of bakery-sourced bread by this group, and whether that bread continues to contain adequately iodised salt, would need further investigation.

### 3.2. Indonesia-Processed Foods and Condiments

Indonesia is undergoing a transformation to an urban economy, with an urbanisation rate of 4.1% per year, faster than other Asian countries, projected to reach 68% by 2025 [6,19,20]. Urbanisation, increasing incomes, and associated changing lifestyles are driving a demand shift in Indonesia and other countries in Asia towards processed foods [6,21]. In parallel, the supply and availability of processed foods are increasing with the expansion of the food processing, procurement and retailing, economic growth and foreign investment, and mass media [22,23]. Indonesia is the world’s second largest per-capita consumer of instant noodles, behind South Korea, with approximately 13.2 billion packets consumed nationally in 2015 [24].

The Indonesian Presidential Decree No. 69 (1994), mandates that all salt for human consumption in the country must be iodised to a level of at least 30 mg/kg potassium iodate (approximately 18 mg/kg iodine). This includes salt for households, the food industry, livestock, salted and curing fish. Regulations required to implement the legislation are in place for household salt (table and cooking salt) with a mandatory Indonesian National Standard (SNI) for salt iodisation. At the time of the research presented below (2013), the Ministry of Industry had not issued any equivalent implementing regulations for iodisation of salt for use in the food industry and no SNI was in place specifically for this type of salt, which was categorised as industrial salt to enable the food industry to import salt of the required food-grade quality.

GAIN analysed data from the Euromonitor Passport 2013 database for Indonesia and worked with PT Nielsen Company, Indonesia, to determine the top five centrally-produced salt-containing foods with highest market penetration. These were: instant noodles, stock (including complete food seasoning), soy sauce, chili sauce and biscuits. Industrially produced bread was also selected for investigation to compare the potential impact of iodised salt in its production with that in other countries. GAIN contracted PT Clarity Research Indonesia to conduct a survey of the major producers of the six identified processed foods and condiments in Indonesia. The purpose was to determine usage of total salt and iodised salt, quality control procedures for salt iodine, and product labelling practices. Out of 45 food producers approached for the survey, 16 agreed to participate, some of these companies produced more than one of the target products. No analysis was conducted into the characteristics of consenting and non-consenting companies, however, the 16 consenting companies together accounted for between 37% and 95% of the market share for their respected food segments (Table 2). It needs to be considered that the findings presented here relate only to the products and percent market share of the product represented by the participating companies. In other words, these data do not necessarily reflect the national situation.

Modelling to estimate the potential contribution of iodine in these products to total dietary iodine intake was based on the equation in the overall methods above, using data and assumptions outlined in the footnote to Table 3.

Most of the companies that participated in the survey used iodised salt, with only two producers (producing three of the target food products) reporting to use non-iodised salt. Among participating companies, approximately 70% of the total salt used in production of selected products was iodised [25]. No information was available to determine whether non-participating companies were using iodised or non-iodised salt.

Two out of the 16 companies interviewed reported the use of a rapid test kit to check that the salt used in their products was iodised. No food producer was measuring the actual iodine content (mg/kg). Most companies relied on the supplier’s Certificate of Analysis (CoA) to determine whether the salt was iodised or not. Ten companies provided the CoA for their last few salt supplies and all of these indicated that salt iodine content was above the national minimum of 18 mg/kg, with six between 18–23 mg/kg and four above 23 mg/kg.

The modelling shown in Table 3 indicates that if only iodised salt was used in the production of the selected products, instant noodles, stock and soy sauce consumption would contribute most to dietary iodine intake at the average estimated per capita intake shown for each product. These three products would account for 6.3%, 3.6% and 2.5% of the adult iodine RNI, respectively. The relative contribution to the RNI for iodine from one serving of each product was significantly higher, at 36%, 20% and 14% respectively. Therefore, among population groups consuming more than one product relatively frequently, it could be expected that these products would be making a significant contribution to total iodine intake. In addition, if salt used in the production had an iodine level of 23 mg/kg, instead of the 18 mg/kg used for the modelling, the relative contribution to iodine intake would be, correspondingly, approximately 1.2 times higher.

The food industry survey found that processed foods known to contribute to salt intake across a range of consumer groups were not consistently produced with iodised salt. However, iodised salt was being used in the majority market share of four of the six targeted products (based only on data from participating companies).

The Indonesian national health research survey in 2013 indicated that only around 55% of the households nationally were using adequately iodised cooking/table salt [26]. However, iodine status among school age children and women of reproductive age at the national and sub-national levels was found to be adequate, implying other possible sources of dietary iodine. These sources could be iodised salt in processed foods together with naturally occurring sources of iodine in foods or ground water. The study reported here indicated that iodised salt was being used in the production of at least 50% of the market share of instant noodles, soy sauce and chili sauce products. These food industry practices could be at least partially responsible for the adequate iodine status observed among some population groups who were not accessing adequately iodised household salt.

The population of Indonesia has been found to have a relatively high level of household expenditure on processed foods in urban and rural areas (respectively, 72% and 64% share of food expenditures was on “high” and “low” processed foods combined) [6]. The proportion spent on “high” processed foods such as those included in this survey (more than one ingredient and requiring a level of processing beyond refining a single product) was higher among urban populations (47% of total processed food expenditure) than among rural populations (36% of total processed food expenditure). Given these data, regulations for the use of iodised salt across the food industry would provide an important contribution to dietary iodine intake.

The two participating companies who did not use iodised salt reported that there was no requirement for them to use iodised salt due to the lack of implementing regulations and that they used non-iodised salt primarily for cost and/or supply chain reasons. However, they would have no objection to switching to use iodised salt if they were legally required to do so [25]. Since the study, a compulsory SNI has been introduced for biscuits (2973:2011 Permenperin No. 60/M-IND/PER/7/2015), however neither this nor the voluntary SNI for instant noodles (3551:2012) specifically mentions the type of salt that should be used in the product. It is, therefore, recommended that implementing regulations are established for food-industry salt, that food product SNIs include specific reference to the use of iodised salt, and that related procedures for enforcement of food industry use of adequately iodised salt are adopted. These steps are in line with the recommendations of a recent review of progress towards sustained elimination of IDD in Indonesia [27] and would provide additional protection from iodine deficiency, in particular among populations that do not have access to adequately iodised household salt.

### 3.3. Philippines-Processed Foods and Condiments

Similarly to Indonesia, the Philippines has seen a rapid expansion of its urban population since 1960, when around 30% of the population lived in urban areas. After an increase to about 49% in 1990, the rate slowed and it is estimated that in 2015 around 44% of the population lived in urban areas [19]. The Philippines has a rapidly expanding food and beverage industry [28] with approximately 90% of industry output consumed domestically [29].

In 1995, the Philippines Congress passed the Act Promoting Salt Iodisation Nationwide (ASIN) [30], mandating iodisation of all salt for human consumption, including salt used by food manufacturers. The Food and Drug Administration (FDA)-authorised standards for iodine levels at production were changed to specify 30–70 mg/kg in 2013 [31]. The FDA has also issued specific standards on the use of iodised salt in food products [32], which allows for exemption where iodised salt has been shown by the food producer to “prejudice” the quality or safety of the food product [33]. At the time of the study described below, there was no available information as to whether food processors complied with the regulations for the use of iodised salt and/or on the amount of adequately iodised salt being used by the food industry. GAIN therefore commissioned the Nutrition Center of the Philippines (NCP) to explore the extent to which iodised salt is used by the processed food industry, and to improve national understanding of dietary iodine sources and industry practices in accordance with the regulations above.

GAIN used the 2008 National Nutrition Survey (NNS) chapter on household food consumption to identify the top five processed foods and condiments contributing substantially to salt intake across population groups, for inclusion in the NCP study. These top five foods from among the 30 products most frequently consumed by all households nationally, were: bread (mainly pandesal, a popular bread roll made of flour, yeast, sugar and salt), soy sauce, instant noodles, crackers, and canned sardines [34]. NCP also included canned corned beef, hotdogs and fish sauce, as food products identified during the study to be contributing to salt intake and produced on a large scale. The study included smaller scale producers of a number of high-salt foods, such as dried fish, fish paste, shrimp paste and sweet pork sausage; however this paper only includes information on findings from major food producers for the five most widely consumed products above, plus canned corned beef, hotdogs and fish sauce. Major producers were defined as those with nationally-recognised brands and at least 8% of the total market share for the product by value.

NCP invited participation from producers of 23 targeted brands of these eight products. Face-to-face interviews were conducted where possible, however two producers requested to self-complete the questionnaire. A 50 g salt sample was collected from each producer visited and tested for iodine content using the iCheck IODINE (BioAnalyt GmbH, Germany) [35] by a trained food technologist.

Typical per capita consumption of each food product was estimated using data from Euromonitor and from the Food and Nutrition Research Institute (FNRI) [28,36], and potential iodine intake from each product was determined based on the formula stated in the overall methods.

The quantity of salt per unit of food product was based on industry-provided data for total salt/total volume of food product produced per batch. The iodine level of salt used to estimate potential contribution to dietary intake was set at 30 mg/kg, according to the national minimum level for iodised salt production.

A total of 11 large food producers, collectively responsible for the production of 13 brands of the targeted products, agreed to participate in the survey. The 13 brands represented the following product type: bread and canned corned beef, one brand each; instant noodles, soy sauce, and fish sauce, two each; canned fish, five; and crackers and hotdogs, none. Some participating producers provided information about more than one of these six products [37].

All 11 producers reported that they were aware of the ASIN law and that they used iodised salt in their production lines for at least some of the surveyed products, ten of these producers included this information on their packaging. With regard to the specific product and brands however, the producer of one brand of fish sauce reported to use non-iodised salt and one brand of instant noodles was produced using both iodised and non-iodised salt. Only six of the eleven producers demonstrated correct understanding of the revised regulated level of iodine in salt (30–70 mg/kg iodine). In terms of checking whether the salt contained iodine or not, 12 brands used the Certificate of Analysis (CoA) submitted by their suppliers to determine that salt was iodised, while the producer of one brand reported to (also) use titration to check the iodine level quantitatively, and salt used in three brands was tested using an in-house semi-quantitative test kit. One product brand was produced using salt that was not checked for iodine content at all.

Eleven of the 13 brands were distributed nationwide, while two were distributed in one region each. Industrially produced bread and canned corned beef were generally bought by consumers of higher socio-economic status, categorised in the Philippines [38] as classes A to C (representing approximately 10% of consumers), while the other products were used by consumers across classes A to D (approximately 70% of consumers) for fish sauce and soy sauce, and classes B to E and A to E for canned fish and instant noodles, respectively.

The results of iodine analysis of six salt samples taken from different production lines of four product types were: low iodine levels (5–10 mg/kg) in one sample of salt used for canned fish production and one sample used for fish sauce production (this was a product reported to include non-iodised salt), between 10 and 30 mg/kg iodine in two products (canned fish and fish sauce), and 30–70 mg/kg iodine for salt from production lines for bread and instant noodles.

The outcome of modelling for each food product is shown in Table 4. Using the more recent food consumption estimates from Euromonitor, the modelling indicates that, if salt iodised at 30 mg/kg iodine was used in the production of these foods, approximately 8 to 10% of the adult RNI for iodine would be met through the average daily per capita consumption of each of: bread, instant noodles, soy sauce, and canned fish, while smaller amounts of iodine would be sourced through the consumption of fish sauce (3.2% of the RNI), hotdogs (3.2%) and canned corned beef (1.2%).

The food industry survey in the Philippines found that among participating producers, processed foods and condiments known to contribute to salt intake across a range of consumer groups were produced using iodised salt. However, the quality of iodisation was only adequate in two of six product lines tested, indicating that company commitment to ensuring that production complied with national regulations on the use of iodised salt varied across the products studied.

The Philippines 8th National Nutrition Survey in 2013–2014 indicated that, nationally, 53% of households were using iodised salt and only 26% of households were using adequately iodised salt [39]. The same survey also reported that, nationally, despite the relatively low use of adequately iodised salt at the household, the overall iodine status was adequate among children 6–11 years of age, adolescents 13–19 years of age, and adults 20–59 years of age. This suggests that, as for Indonesia, there were likely to be other dietary sources of iodine contributing to the improvement, some possibly from the use of iodised salt in certain processed foods. However, iodine status was not adequate among lactating and pregnant women and among the elderly. It was also inadequate among children 6–11 years of age living in households categorised to be in lowest two wealth quintiles.

The reported food industry practices could be at least partly responsible for the observed improvement in iodine status among children 6–11 years of age from a level of deficiency in 1998 [40], during which time there has been little change in household coverage with adequately iodised salt. However, it is clear that these practices also require strengthening in line with national regulations. Increased attention to quality control by the producers and to regulatory monitoring on the part of the government would provide greater certainty of additional iodine intake from this source, including among currently deficient groups such as pregnant women and the elderly.

### 3.4. Ukraine and the Russian Federation-Bread

The dissolution of the Soviet Union in 1991 resulted in the absence of a legislative framework to support regulatory monitoring of iodised salt production. Most post-Soviet countries have since adopted national USI strategies [41], however, despite clear evidence of widespread deficiency in their populations, the governments in the Russian Federation and Ukraine have been reluctant to re-introduce mandatory USI, interpreting it as an alleged potential violation of “consumers’ right for choice” and “industry’s freedom of enterprise” [41,42]. The Government of Ukraine adopted a decree in 1997 and the Government of the Russian Federation adopted a resolution (No. 119) in 1999, both titled “On measures to prevent iodine deficiency disorders”. However, the associated regulations in both decrees stipulated a voluntary model of prevention, with no specific enforcement mechanism in place and resulted in low levels of production and supply of iodised salt in both countries [43].

Bread is a staple food in both countries, with average annual per capita consumption estimates of about 86 kg (Ukraine) and 100 kg (Russian Federation) [44,45] with variations between regions and rural and urban areas. Given the political resistance to USI, the Partnership Project hypothesised that a narrower, specifically tailored mandate of using iodised salt in bread baking and in public catering institutions, an effective tactic in countries including the Netherlands, Belarus, and Australia [42,46,47], could be more acceptable to policy makers than iodising the entire market supply of salt. In a 2010 poll by the Russian Union of Bakers of over 100 bread factories from 25 regions, >90% of bread factories reported producing small volumes of bakery products enriched with iodine, the majority used an iodised protein-based fortificant, with only 5% using iodised salt. Of the responding bread companies, 27% expressed objections to iodised salt, citing risk of iodine “overdose”, perceived loss of iodine from the “wet” storage of salt prior to addition to the dough, and low perceived stability of iodised salt in dough during baking [48]. In Ukraine, a 2012 review of barriers to using iodised salt in bread baking highlighted that while existing regulations did not prohibit the use of iodised salt, they also did not explicitly permit it [49]. About a third of producers surveyed believed that iodine would evaporate during bread baking, with other major concerns related to an increase of costs and the absence of demand among consumers. Only 4% of producers surveyed in Ukraine used iodised salt in some specific types of bread.

The GAIN-UNICEF USI Partnership Project initially engaged in high level advocacy with key policy makers in both countries aiming for mandatory legislation on the use of iodised salt in bread bakeries. Specific activities included appeals by the UNICEF Goodwill Ambassador in Russia, support for the preparation of draft legislation put before the Council of Ministers in Ukraine, and hosting of a high level regional forum in 2011 in Belgrade, Serbia, which brought policymakers, academics, consumer interest groups and the food and salt industries from both countries together with regional champions for USI from Belarus, Kazakhstan, and Serbia [50]. However, because these activities did not result in any shift in policy in Ukraine or the Russian Federation, the Partnership Project changed its strategy toward promoting the voluntary use of iodised salt in the manufacture of bakery products, which is permitted within current legislation in bread production in both countries. The Partnership Project approach was to sensitise bread producers and remove real and perceived barriers within the bread industry through the development of an enabling environment to adopt iodised salt use as an industry norm. A Bread Forum was held in Lviv in 2012 to address these issues.

In the Russian Federation, the Partnership Project supported the Moscow University of Technologies and Management to draft recommendations on the use of iodised salt in bread. To address bakers’ technical concerns, The Partnership Project supported the Research Institute of the Baking Industry, the body responsible for setting baking regulations, to conduct operational research on the use of iodised salt in the production of various types of Russian bread. In line with international literature, the research confirmed 70% retention of iodine and a lack of negative effects on the bread’s taste or quality. In contrast, baking with iodised salt was shown to provide several benefits including increased stability, porosity, and volume of the final bread product [48]. These results were published in Russian in professional bakery journals and presented in national and regional industry workshops (S. Kulev, GAIN consultant, personal communication, 2013).

In Ukraine, the Partnership Project focused primarily on building consumer demand and bread industry approval for iodised salt use in bread products through an awareness campaign and evidence of success of this approach in other countries. The Partnership Project liaised with the Bread Association to disseminate the Russian research and brought technical experts from the Russian and Belarussian bread industry to conduct interactive workshops with Ukrainian bakers. Also a national public health communication campaign on iodine was conducted, and an official iodised salt logo was made available for bakeries and other food processors for use on their products. The campaign promoted the logo on billboards, at urban and rural food fairs and on city light displays, posters, booklets, and leaflets attracting over 6 million views over 2012–2013 [51]. Finally, the Partnership Project consulted with individual bread companies to understand and overcome their perceived barriers to using iodised salt in bread products, through provision of technical and advocacy support, including providing testing equipment to measure iodine levels in salt in their factories.

The approach to convince bread industries in Russia and Ukraine to voluntarily use iodised salt in bread products purchased across a wide-spread consumer base did not achieve its objective. Although the Partnership Project was successful in garnering support from among some industry stakeholders for mandatory legislation for the use of iodised salt in bread production [52], the technical assistance, education, and demand creation activities undertaken were ultimately insufficient to bring about change in government and industry attitudes and norms. In the absence of mandatory legislation, bread producers were generally unwilling to take the risk of switching to iodised salt in production of common “social” bread because they considered “iodised bread” to be a special “functional” product. Without clear policy direction, confusion remained about whether using iodised salt would require a change in official recipes and associated documentation and be subject to costly labelling and quality control procedures [49].

In the Russian Federation, several large bread companies switched to using iodised salt instead of other, more expensive, iodine fortificants for fortified “functional” bread brands after attending the Partnership Project’s industry workshops (S. Kulev, GAIN consultant, personal communication, 2013). However, these special bread products do not generally reach the majority of the population, including groups with potentially less diverse diets and lower access to other sources of dietary iodine.

The communication and awareness-raising activities of UNICEF and GAIN in Ukraine resulted in two registered medium- to large-scale bakeries located in Lviv and Kiev changing their production processes to use iodised salt in bread baking in 2012. The volume of bread production reported by these bakeries had an estimated reach of 19,000 people (about 0.04% of the population), based on an average per capita consumption of 240 g bread/day [45].

Modelling of potential adult iodine intake from the use of iodised salt in all bread products is shown in Table 5. Modelling was based on information provided by these two bakeries and on the industry research showing approximately 70% retention of iodine from salt in the final product.

Bread intake is relatively high in Ukraine and in the Russian Federation, and the salt iodisation standard in both countries is also relatively high, at 40 mg/kg ± 15 mg/kg. As a result, it was found that if adults were assumed to be consuming only bread from bakeries where all the bread was iodised at the minimum acceptable level of 25 mg/kg, they would be sourcing approximately 32% (Ukraine) and 37% (Russian Federation) of the adult RNI for iodine through bread consumption.

Iodine deficiency has been clearly demonstrated among the national population of reproductive age women in Ukraine [53], particularly associated with households using non-iodised or inadequately iodised salt [43]. Similarly, data from regions within the Russian Federation during the period 2002–2006 showed that the population in 16 out of 19 regions surveyed were iodine deficient [54]. Despite this evidence of deficiency and an awareness of the related implication for decreased population IQ through impaired foetal and child brain development, there is no political will to legislate for mandatory iodisation of cooking/table salt in either country. The alternative suggested approach was to mandate for the use of iodised salt in the production of “social” bread, which reaches a wide cross section of the national populations and would provide approximately one third of the daily adult RNI for iodine. This level of iodine intake would be expected to improve total intake of the population to a level of iodine sufficiency.

The reported technical assistance and advocacy efforts did improve the baking industry’s stated acceptance of the need to use iodised salt, however, this did not translate into changed industry practice during the time of the Partnership Project. Reasons for this may have included: (a) the concerns raised among bread producers in the above-cited surveys may not have been the actual reasons for not using iodised salt; (b) the Partnership Project was operating during a time of serious economic downturn, which generally discourages anything perceived as a potential risk to industry performance; and (c) a general practice of respect for and compliance with government-directions, which were not strongly supportive of the initiative.

An informal pro-USI coalition (Public Coordination Council) together with other partners in the Russian Federation continue to raise public awareness about iodised salt. The Public Coordination Council is actively lobbying the government to adopt an amendment to the public health law, developed by the Department of Public Health under the Ministry of Health, which would mandate iodisation of certain types of household salt and the use of iodised salt by the bread baking industry [52]. Additional analyses of the policy and bakery industry situation would be required in Ukraine to assess the value of renewed advocacy there also.

## 4. Discussion

These case studies in five countries demonstrate that the use of adequately iodised salt by the food industry can make a significant contribution to dietary iodine intake. The findings show that, to achieve the intended impact of salt iodisation on iodine status, legislation needs to mandate iodisation for all food-grade salt and be accompanied by clear regulations for implementation, with designated responsibilities and procedures for monitoring and enforcement of the use of adequately iodised salt within the food industry.

The work in the Russian Federation and Ukraine demonstrated the difficulty of trying to generate large-scale industry change to use iodised salt on a voluntary basis. This finding is consistent with lessons from other large-scale fortification initiatives [55,56].

In other countries, factors found to influence compliance with legislation by food industries included: the size of the company, the industry’s awareness of the legislation, regulatory loopholes in the legislative framework, prescribed salt standards for the specific food product, access to adequately iodised salt supplies, and government practices in regard to regulatory monitoring. A 2015 study of barriers and good practices in regulatory monitoring of fortified foods in LMIC found similar lessons that applied across a range of fortified products [57]. The conclusion of that study was that the full intended health benefit of a food fortification strategy will only be achieved where the entire regulatory system is built on a cooperative working relationship between regulatory agencies and food producers.

In countries with USI legislation, a recommended strategic priority is to strengthen monitoring and enforcement of iodisation of salt for large-scale, registered food industry use, at the point of salt production or import and at the food production site. In particular, an initial focus on products widely consumed across population groups would be both feasible and likely to have a meaningful impact on population iodine status.

As consumption patterns and dietary sources of salt often differ between different segments in a population, it is important that all food-grade salt is iodised at a level appropriate to achieve and sustain optimal iodine status across the population. For example, in Egypt, the estimated levels of iodine in salt used by bakeries in different governorates (Table 1) varied from 16.5 mg/kg in Beni-Suef to 57.3 mg/kg in Kalyubia, meaning that consumers with a high intake of Baladi bread in Kalyubia may have been meeting the RNI for iodine from Baladi bread alone. Establishing national salt iodine standards based on estimates of total salt intake, and regulating these levels across all dietary sources of salt will help achieve adequate iodine status and prevent the risk of excessive iodine intake due to high consumption of a particular food.

Enforcement of regulations is typically more challenging within artisanal industries which may be more difficult to locate and/or have limited financial and technical capacity to comply with required standards [57,58]. However, the case study in Egypt with many small-scale bakeries provides an example where this is not necessarily the case. All bakeries in Egypt are required to be registered in order to qualify for government-subsidised wheat flour, which made it relatively easy for inspectors to list, locate and inspect the bakeries for the type of wheat flour and salt used. The food producer study conducted by NCP in the Philippines included a range of artisanal producers of bread, sweet pork sausage, salted fish and shrimp paste products (not reported here). Iodine tests of the salt used in this artisanal segment showed that about three quarters of 144 product lines were using non-iodised or inadequately iodised salt [37]. This suggests that additional strategies will be required to ensure that artisanal producers are informed of, and able to comply with, national standards where these exist. Monitoring thousands of artisanal food producers would be resource-intense, however. One approach may be to ensure monitoring of the general market supply of consumer salt at the source (i.e., salt production and import sites), which is often the most likely origin of salt for small food producers.

The current, and increasing, importance of food industry salt in total dietary salt intake has been emphasised in a number of papers [3,4,6,7], as well as in the case studies reported here. Assessing the success of a true USI strategy, which includes iodisation of food industry salt, will require indicators and monitoring strategies in addition to the currently recommended indicator of adequately iodised household salt coverage [13]. An example would be the estimation of food industry salt iodine intake from knowledge of food industry iodised salt use in combination with population food frequency estimates and/or detailed consumer surveys. When dietary iodine from all sources of salt is estimated (qualitatively and biochemically) alongside population iodine status surveys, it will be possible to better determine whether dietary iodine intake as a result of a national USI strategy leads to overall optimal iodine status or whether the salt iodine standard should be adjusted.

## 5. Conclusions

The food industry’s potential contribution to dietary iodine intake through the use of iodised salt in food processing is significant and of growing importance. However, in many countries, food industry salt has not been a major focus of national USI strategies and, even where regulations exist, regulatory monitoring of food industry salt has not usually been practiced. Moving forward it is recommended that: the food industry is included in advocacy and communication about USI to ensure they are informed of the need and industry expectations; legislation for the use of adequately iodised salt by the food industry is developed or strengthened with clear regulations and enforcement mechanisms and penalties; consistent access to adequately iodised salt is ensured; and the contribution of dietary iodine from food industry salt is monitored and evaluated. These recommendations can be implemented in coordination with salt reduction strategies, ensuring that the iodine level of food grade salt is appropriate for average overall salt intake [59]. International guidance on how these steps can be included in national programs for the achievement of optimal iodine nutrition have recently been developed and/or are in the final stages of development [60,61]. Much of this guidance has been developed as part of the Partnership Project and/or based on lessons learned during the project, such as these case studies above.

## Figures and Tables

**Table 1:** Results of modelling the potential dietary intake of iodine from bakery-produced Baladi bread in Egypt.

			Mean Estimates by Governorate			
Region	Governorate	Number Samples/Governorate	Measured Iodine in Bread µg/100 g Bread	Derived Iodine Content (mg/kg) of Bakery Salt	µg Iodine Daily from Intake 400 g Bread ^1^	% RNI ^2^ for Iodine
	National	207	18.9	36.0	75.6	50.4%
Metropolitan	Cairo	10	21.9	48.9	87.6	58.4%
	Alexandria	10	12.1	23.8	48.4	32.3%
	Port Said	2	12.0	21.0	48.0	32.0%
	Suez	11	13.5	26.1	53.8	35.9%
Lower Egypt	Sharkia	10	26.3	48.8	105.2	70.1%
	Menoufia	6	9.7	17.7	38.4	25.6%
	Gharbia	4	9.5	17.0	38.0	25.3%
	Damietta	10	18.1	35.4	72.4	48.3%
	Dakahlia	3	20.7	35.1	82.7	55.1%
	Kafr El-Shiekh	9	22.3	44.2	89.3	59.6%
	Ismailia	9	18.9	35.9	75.6	50.4%
	Kalyubia	10	29.8	57.3	119.2	79.5%
Upper Egypt	Aswan	10	21.4	37.7	85.6	57.1%
	Qena	10	15.4	28.3	61.6	41.1%
	Luxor	10	24.5	48.3	98.0	65.3%
	Souhag	10	12.9	24.0	51.6	34.4%
	Assuit	10	27.3	52.7	109.2	72.8%
	Giza	9	23.6	41.7	94.2	62.8%
	Fayoum	15	13.9	25.4	55.7	37.2%
	Beni-Suef	9	9.1	16.5	36.4	24.3%
	Menya	6	23.0	48.1	92.0	61.3%
Frontier	Red Sea	6	25.0	46.5	100.0	66.7%
	South Sinai	8	17.5	34.2	70.0	46.7%
	Matrouh	5	22.6	41.2	90.4	60.3%
	New Valley	5	13.6	25.3	54.4	36.3%

^1^ Conservative estimate of typical adult bread intake (380 g/day flour equivalent to 475 g/day bread [10]) and communication with Dr. Magdy Shehata, WFP Egypt Programme Officer (350 g flour/day equivalent to 438 g bread). ^2^ RNI, recommended nutrient intake (iodine) is 150 µg for an adult [13].

**Table 2:** Overview of food industry survey participants, food products and market share, and use of iodised salt, Indonesia.

Product	Number of Companies Participating in Survey	Market Share of Participating Companies	Participating Companies Using Iodised Salt	Market Share of Participating Companies Using Iodised Salt ^1^
Instant noodles	6	67%	5	66%
Stock	2	37%	1	8%
Soy sauce	4	82%	3	51%
Chili Sauce	6	94%	6	94%
Biscuits	3	38%	3	38%
Industrial bread	2	95%	2	95%

^1^ Percentage market share using iodised salt based on market share of participating companies multiplied by the percentage of salt used by these companies that was iodised.

**Table 3:** Potential adult iodine intake from selected products in Indonesia if all salt used in production is iodised at the level of 18 mg/kg iodine.

Food Product	Estimated Average Serving Size (g) ^1^	Equivalent Salt Intake from One Serving Size of the Product (g)	Potential Iodine Intake from One Serving Size of the Product (µg) ^2^	% Daily RNI for Iodine from One Serving Size of the Product (µg) ^3^	Estimated Average Annual per Capita Consumption of the Product (g)	Estimated Minimum per Capita Daily Iodine Intake from the Product (µg) ^2^	% Daily RNI for Iodine
Instant noodles ^4^	85	3.0	54	36%	5444	9.5	6.3%
Stock	3	1.6	29	20%	200	5.3	3.6%
Soy sauce	15	1.2	22	14%	940	3.7	2.5%
Bread ^5^	Cannot calculate from available data				1900	1.4	0.9%
Chili sauce	5	0.2	4	3%	340	0.7	0.5%
Biscuits ^6^	25	0.5	8	6%	1300	1.2	0.8%
					300	0.3	0.2%

^1^ Serving sizes based on data from Euromonitor Passport 2013, World Noodle Association, Mintel Global Market Navigator, and Clarity survey findings. ^2^ Iodine intake based on 18 mg iodine per kg salt according to the national standard for minimum iodine content. ^3^ Recommended nutrient intake (RNI) for iodine for an adult is 150 µg [14]. ^4^ Estimate for g salt per serving size (85 g pack) of noodles is based on a combination of information from Clarity-surveyed producers and Ministry of Industry data for the amount of salt used in instant noodle production, compared with the number of packets consumed per year (World Noodle Association) and the UNICEF-supported universal salt iodisation (USI) Programme Review (which suggested 3.2 g salt/pack of noodles). ^5^ It was not possible to calculate the estimated serving size from data obtained during the survey however one of the largest bread manufacturers, PT Nippon Indosari Corpindo Tbk estimated an annual per capita intake of 1900 g bread in 2012 [25], which was used to estimate potential iodine intake from bread if all salt used in its production was iodised. ^6^ Two different options presented for biscuits according to annual per capita consumption data from Mintel Global Market Navigation and Roy Morgan, estimating 1.3 kg/pers/year; and from interviews with surveyed biscuit producers, estimating 0.3 kg/pers/year.

**Table 4:** Potential adult iodine intake from selected products if all salt used in production is iodised at the level of 30 mg/kg iodine, the Philippines.

Food Product	Gram Salt/Gram Food Product	FNRI Daily per Capita Intake (g)		Euromonitor Daily per Capita Intake (g)		Potential Iodine Intake (µg) from Average Daily per Capita Intake ^1^		% of Adult RNI for Iodine ^2^	
		Food Product	Related Salt Intake	Food Product	Related Salt Intake	FNRI	Euromonitor	FNRI	Euromonitor
Bread	0.04	11.0	0.4	13.4	0.5	11.9	14.5	7.9	9.7
Instant noodles	0.09	4.0	0.4	4.9	0.5	11.2	13.8	7.4	9.2
Canned fish	0.11	8.0	0.9	3.63	0.2	26.9	12.0	17.9	8.0
Soy sauce	0.13	3.0	0.4	3.0	0.4	11.8	11.8	7.9	7.9
Fish sauce	0.59	ND	ND	0.3	0.2	ND	4.8	ND	3.2
Canned corned beef	0.03	ND	ND	1.94	0.1	ND	1.8	ND	1.2

FNRI, the Food and Nutrition Research Institute. ND = no data. ^1^ Based on salt iodine content of 30 mg/kg. ^2^ RNI for iodine for an adult is 150 µg [14].

**Table 5:** Potential adult iodine intake from bread if all salt used in bread baking is iodised at the level of 25 mg/kg iodine, Ukraine and the Russian Federation.

Food Product	Country	Estimated Daily per Capita Intake (g)	Estimated Salt Content per Gram of Bread ^1^ (g)	Average Daily per Capita Salt Intake from Bread (g)	Estimated per Capita Daily Iodine Intake from Bread (µg) ^2^	% Daily RNI for Iodine ^3^
Bread	Ukraine	240	0.012	2.7	48	32%
Bread	Russian Federation	280	0.012	3.2	56	37%

^1^ Based on 2012 reports from two bakeries in Kiev and Lviv, Ukraine. ^2^ Assuming all salt used in bread production contains 25 mg iodine/kg of which 70% (17.5 mg) is retained. ^3^ RNI for iodine for an adult is 150 µg [13].
